# *In situ* counter-diffusion crystallization and long-term crystal preservation in microfluidic fixed targets for serial crystallography

**DOI:** 10.1107/S1600576724007544

**Published:** 2024-09-25

**Authors:** Zhongrui Liu, Kevin Gu, Megan Shelby, Debdyuti Roy, Srinivasan Muniyappan, Marius Schmidt, Sankar Raju Narayanasamy, Matthew Coleman, Matthias Frank, Tonya L. Kuhl

**Affiliations:** a Department of Materials Science and Engineering University of California Davis Davis CA95616 USA; b Department of Chemical Engineering University of California Davis Davis CA95616 USA; c Biosciences and Biotechnology Division Lawrence Livermore National Laboratory Livermore CA94550 USA; d Biophysics Graduate Group University of California Davis Davis CA95616 USA; e Physics Department University of Wisconsin–Milwaukee 3135 North Maryland Avenue Milwaukee WI53211 USA; f Department of Radiation Oncology, School of Medicine University of California Davis Sacramento CA95817 USA; g Department of Biochemistry and Molecular Medicine, School of Medicine University of California Davis Sacramento CA95817 USA; Uppsala University, Sweden; The European Extreme Light Infrastucture, Czechia

**Keywords:** serial crystallography, vacuum operation, protein diffraction, X-ray scattering, XFELs, counter diffusion, *in situ*, long-term crystal preservation, microfluidic fixed targets

## Abstract

Compared with batch and vapor diffusion methods, counter diffusion can generate larger and higher-quality protein crystals yielding improved diffraction data and higher-resolution structures. Typically, counter-diffusion experiments are conducted in elongated chambers, such as glass capillaries, and the crystals are either directly measured in the capillary or extracted and mounted at the X-ray beamline. Despite the advantages of counter-diffusion protein crystallization, there are few fixed-target devices that utilize counter diffusion for crystallization. In this article, different designs of user-friendly counter-diffusion chambers are presented which can be used to grow large protein crystals in a 2D polymer microfluidic fixed-target chip. Methods for rapid chip fabrication using commercially available thin-film materials such as Mylar, propyl­ene and Kapton are also detailed. Rules of thumb are provided to tune the nucleation and crystal growth to meet users’ needs while minimizing sample consumption. These designs provide a reliable approach to forming large crystals and maintaining their hydration for weeks and even months. This allows ample time to grow, select and preserve the best crystal batches before X-ray beam time. Importantly, the fixed-target microfluidic chip has a low background scatter and can be directly used at beamlines without any crystal handling, enabling crystal quality to be preserved. The approach is demonstrated with serial diffraction of photoactive yellow protein, yielding 1.32 Å resolution at room temperature. Fabrication of this standard microfluidic chip with commercially available thin films greatly simplifies fabrication and provides enhanced stability under vacuum. These advances will further broaden microfluidic fixed-target utilization by crystallographers.

## Abstract 2

User-friendly *in situ* counter-diffusion crystallization and long-term storage setups in microfluidic fixed targets provide a reliable approach to forming large crystals and maintaining their hydration for weeks, allowing ample time to grow, select and preserve the best crystal batches before X-ray beam time.

## 1. Introduction

X-ray crystallography has been the primary method for protein structure determination for several decades. Light-source facilities, such as synchrotrons and X-ray free electron lasers (XFELs), provide a wide range of beam energies and pulse durations to accommodate various crystallography experiments. XFELs are especially powerful due to their high intensity and ultrashort X-ray pulses, allowing for efficient diffraction data collection from small crystals on a ‘diffract before destroy’ basis (Aquila *et al.*, 2012 ▸; Patterson, 2014 ▸). However, access to these powerful XFELs is limited, as they are highly over-subscribed. The recent development of a compact XFEL (CXFEL), a room-sized facility at much lower cost, will increase access to ultrashort pulses for more researchers and institutions (Rosenzweig *et al.*, 2020 ▸; Malin *et al.*, 2018 ▸). However, the CXFEL has a much lower brilliance compared with XFELs, making it more suitable for repetitive measurements on a large protein crystal, instead of merging diffraction from numerous tiny individual crystals with serial crystallography approaches like at traditional XFEL beams. Likewise, synchrotron beamlines can benefit from large and high-quality crystals for improved diffraction data (Clegg, 2019 ▸).

The three main crystal sample-delivery methods for X-ray facilities are fixed targets, liquid and lipid cubic phase (LCP) injectors, and tape drives (Hunter *et al.*, 2014 ▸; Weierstall *et al.*, 2014 ▸). Liquid jets and tape drives involve pumping crystal slurries, which can lead to challenges such as crystal clogging and difficulties in controlling the size of the slurry stream or droplet, as well as high consumption of crystal slurry (Konold *et al.*, 2023 ▸; Zielinski *et al.*, 2022 ▸). These methods are not suitable for large crystals. While LCP injectors provide more control and protection for membrane crystals with little sample waste, the resolution can be limited. In contrast, fixed targets are robust and flexible. They can handle large and small crystals, are compatible with LCP samples, minimize the need for manual intervention, offer more options for sample storage, and can be customized to match specific beamline requirements including vacuum operation.

By controlling nucleation and promoting crystal growth, counter diffusion is a technique known for producing larger and higher-quality crystals than batch and vapor diffusion methods (Otálora *et al.*, 2009 ▸). Protein crystallization involves controlled manipulation between supersaturated, meta-stable and stable phase states. By adjusting temperature and concentration, the system is guided through the phase diagram to form and grow crystals. While supersaturation is necessary for initiating crystallization, straying too far from equilibrium can result in very small crystals with a high level of impurities. To cultivate large high-quality crystals, it is vital to regulate the driving forces to ensure a gradual and consistent supply of essential masses at the crystal-growth front, as well as keeping the dynamic equilibrium of the system. Since complete control over kinetics in a protein-crystallization setup is unattainable (Petsev *et al.*, 2003 ▸), most efforts focus on reducing convective mass transport in the system so that diffusion of proteins and precipitating agents dominates. Rather than inducing nucleation by rapidly hitting supersaturation through vigorously mixing proteins and precipitating agents like in batch or vapor crystallization methods, diffusion-controlled transport gradually introduces the precipitating agent. Controlling mass transport and aggregation kinetics is crucial in counter-diffusion setups (Ng *et al.*, 2003 ▸; García-Ruiz, 2003 ▸; Otálora *et al.*, 2009 ▸).

To grow large crystals, one would ideally design a system with pure diffusion, which can be approached in a microgravity environment (Zegers *et al.*, 2006 ▸) or by using other approaches that minimize convection and gravity effects such as high-sample-viscosity solutions (Tanaka *et al.*, 2022 ▸), hydro­gels (Gavira *et al.*, 2020 ▸), capillaries (Maes *et al.*, 2006 ▸) and microfluidic devices (Hansen *et al.*, 2002 ▸; Dhouib *et al.*, 2009 ▸). These approaches are used in counter diffusion to maintain low nucleation rates and slow crystal growth, thereby enhancing crystal quality. Nevertheless, many successful counter-diffusion setups still involve harvesting crystals or collecting data through thick glass capillaries (Kober *et al.*, 2023 ▸; González-Ramírez *et al.*, 2017 ▸; Kurz *et al.*, 2012 ▸). Counter diffusion in fixed-target chips allows *in situ* crystallization and beamline measurements in a single device. Saha *et al.* (2023 ▸) fabricated an entire chip with SU-8 and utilized centrifuge loading to efficiently set up counter-diffusion trials. A microfluidic device called ChipX (de Wijn *et al.*, 2021 ▸) also utilized counter-diffusion techniques to grow crystals *in situ* in a microfluidic chip for serial crystallography measurements with separated crystallization buffer channels and a convenient single inlet for the protein solution. However, these chips have relatively thick polymer windows that the X-ray beam must pass through, resulting in high background and lower resolution. Furthermore, these chip designs require expensive microfabrication steps that limit design flexibility.

In this work, we present a microfluidic chip and counter-diffusion methods that offer a cost-effective and reliable method for producing large crystals and preserving their hydration for extended periods, lasting weeks or even months. This extended sample stability allows ample time for users to grow, select and preserve the best crystal batches prior to their beam time. Furthermore, the microfluidic chip containing the grown crystals can be directly used for measurements without requiring further manipulation, making it very convenient to use a single device for both crystallization and X-ray diffraction measurements, thereby maintaining crystal quality. The microfluidic chip has been previously demonstrated to yield low background, and high-quality diffraction data were obtained (Liu *et al.*, 2023 ▸). Here, we demonstrate counter-diffusion crystallization under various conditions using lysozyme and measurements of high-resolution low-background diffraction with photoactive yellow protein (PYP). We also detail some simple modifications to improve vacuum stability and rapid fabrication using commercially available X-ray compatible polymer films. The chip’s user-friendly nature and ability to store the best batches for weeks, and the ease of handling fixed-target chips, will be valuable additions to the toolkit of structural biologists.

## 2. Experimental setup

Cyclic olefin copolymer (COC; TOPAS Advanced Polymers Grade 6017, Tg = 170°C) was purchased from Polysciences Inc., USA. Polyvinyl alcohol (PVA) was purchased from Sigma–Aldrich (Product 363170). Sec-butyl­benzene (99.0%, TCI America, catalog No. B0714500ML) was used as the solvent to dissolve the COC. Mylar window film (3 µm, EW-04575-98, Spex BoPET XRF Window Film) was purchased from Cole-Parmer. Ultrathin Kapton HN polyimide (5 µm) was purchased from PolyK. Kapton 50EN thin film of 12.5 µm was purchased from DuPont. As a supporting frame material, 1 mm-thick transparent polymethyl methacrylate (PMMA) sheets (SimbaLux, 5″ × 7″) were purchased from Amazon. Double-sided acrylic adhesive was purchased from Adhesives Research (ARcare 92712) and adhesive transfer tape was purchased from 3M (F9460PC). Storage chambers were cut from 18″ × 24″ × 0.093 (3/32)″ clear acrylic sheets from OPTIX, purchased from the Home Depot. Clear silicone rubber gasket sheet (LMS, 12″ × 19.7″ × 1/25″) and Weld-On 4 acrylic adhesive were purchased from Amazon. Chicken egg-white lysozyme (catalog No. L6876) was purchased from Millipore Sigma (St Louis, Missouri, USA). Pluronic F-127 was purchased from Sigma–Aldrich (P2443) to make hydro­gel. PYP was purified and provided by Srinivasan Muniyappan and Marius Schmidt at the University of Wisconsin–Milwaukee, on the basis of the work of Tenboer *et al.* (2014 ▸), Kort *et al.* (1996 ▸), Imamoto *et al.* (1995 ▸), Borgstahl *et al.* (1995 ▸) and Meyer (1985 ▸).

### 2.1. Chip and chamber fabrication

**Figure 1:**
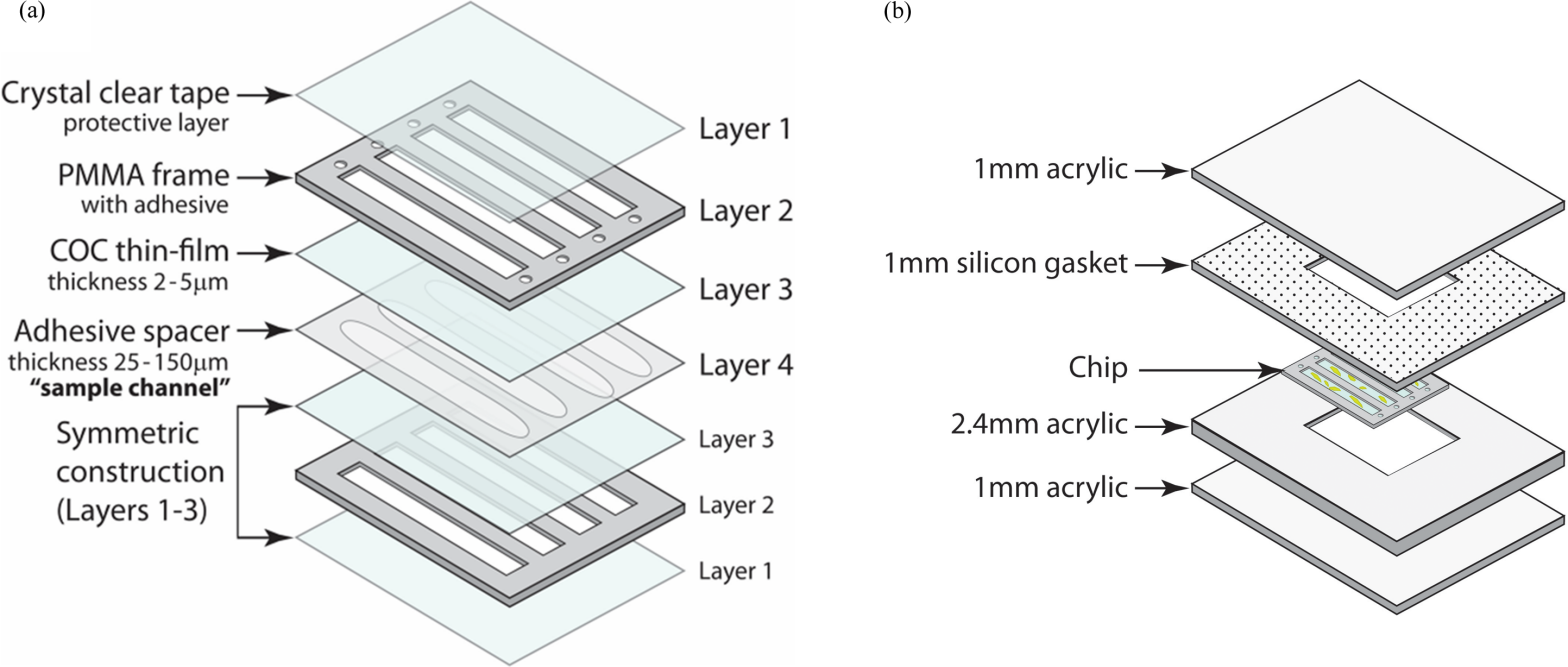
(*a*) A schematic drawing of the all-polymer fixed-target chip construction layers, adapted from Gu *et al.* (2023 ▸) with the permission of AIP Publishing. There are two crucial features of the design. First, a CO_2_ laser is used to cut the adhesive spacer that controls the sample thickness (layer 4) and the thick supporting PMMA frame with double-sided adhesive on one side (layer 2). These layers establish the geometry of the fixed-target chip. The thin films that enclose the sample and through which the X-ray beam passes (layer 3) can be fabricated out of various polymer thin films. The system is completely tailorable to meet user sample and beamline requirements. Layer 1 is a removable protective cover layer of Crystal Clear tape that prevents window film damage and sample dehydration. (*b*) A 3D schematic drawing of a fixed-target chip in the customized acrylic storage chamber. The chamber design allows for direct imaging during crystallization without having to open the chamber. The indent on the acrylic chamber was designed to snugly fit the chip to minimize the free volume that the sample must equilibrate with. Less than 1% sample volume/weight change is needed to equilibrate between 0 and 100% relative humidity conditions (Gu *et al.* 2023 ▸).

As described in our recent publication (Liu *et al.*, 2023 ▸), the microfluidic chip is assembled from four different polymer layers, as shown in Fig. 1 ▸. The enclosed X-ray imaging areas can be made from pre-fabricated thin films such as Mylar, Kapton or COC [Fig. 1 ▸(*a*), layer 3]. Our work has primarily used 2–3 µm COC thin films due to their low background scatter and high stability during XFEL measurements (Liu *et al.*, 2023 ▸). Commercial Mylar and Kapton thin films improve vacuum compatibility but are not suitable for XFEL measurements (Doak *et al.*, 2018 ▸; Lee *et al.*, 2019 ▸; Murray *et al.*, 2015 ▸). The COC thin films were prepared by spin-coating solutions of COC onto silicon wafers (Liu *et al.*, 2023 ▸; Gu *et al.*, 2023 ▸; Gilbile *et al.*, 2021 ▸). The solutions were prepared by dissolving 15 wt% COC in *sec*-butyl­benzene at 120°C overnight or until fully dissolved. Control over film thickness from 500 nm to 5 µm is possible depending on the spin speed and solution concentration. To improve the delamination of the COC thin film from the silicon wafer, a water-soluble sacrificial layer of 9 wt% PVA in Milli-Q water was first spun onto a clean UV–ozone-treated silicon wafer before COC film deposition. A 15 min UV–ozone treatment was used to improve the surface wettability of the silicon wafer. The PVA sacrificial layer was baked on a hotplate at 120°C for a few minutes to evaporate residual water. Afterwards, warm COC solution (>80°C) was spun on top of the dried PVA layer at 1000 r min^−1^ for 60 s to obtain a 2–3 µm-thick film. The thin film was supported by a stiff PMMA frame. A piece of double-sided adhesive transfer tape was attached to the PMMA sheet before laser cutting. After laser cutting, the adhesive backing was removed and the laser-cut PMMA frames [Fig. 1 ▸(*a*), layer 2] were adhered to the silicon-wafer-supported COC film to create a half-chip. The wafer with attached frames was then placed in water to release the half-chip. Scoring with a razor after applying layer 2 dramatically decreases the time for film release. Afterwards, a laser-cut spacer [Fig. 1 ▸(*a*), layer 4] was attached to one of the half-chips. Before assembling the two half-chips to make a complete chip, the inlet holes are punctured with tweezers. The chips can be prepared months in advance. Placing a weight on top of the assembled chips overnight can improve the adhesive seal between the various layers after assembly.

To use the chip, a sample is simply pipetted into an assembled chip or loaded prior to adhering the two chip halves for vapor-diffusion, microbatch or counter-diffusion crystallization. For vapor diffusion, the entire chip can be placed into a controlled environment for crystallization. In the case of microbatch crystallization, after loading the sample(s), a piece of removable Crystal Clear tape [Fig. 1 ▸(*a*), layer 1] is placed over the PMMA frames, covering the entire window and the inlets without touching the thin film on the window. This setup protects the samples from dehydration. The tape can be easily removed with a razor immediately before mounting the sample at the beamline, exposing the X-ray imaging windows while keeping the inlets covered during the measurements (Liu *et al.*, 2023 ▸; Gu *et al.*, 2023 ▸; Gilbile *et al.*, 2021 ▸).

The various counter-diffusion setups [Fig. 1 ▸(*b*)], which also serve as storage chambers, were designed to be compatible with our all-polymer microfluidic chips [Fig. 1 ▸(*a*)]. The customized acrylic chambers were fabricated by laser-cutting clear acrylic sheets and a silicone rubber gasket layer. The chamber was designed to snugly fit the chip dimensions, minimizing the volume equilibrating with the solution in the microfluidic channel [Fig. 1 ▸(*b*)]. Minimizing the free volume around the chip minimizes the volume that the chip will equilibrate with during long-term storage, resulting in <1% hydration change from 0 to 100 relative humidity. For a 1″ × 1″ chip with 1 mm acrylic support on both sides, a piece of 2.4 mm acrylic was glued onto a 1 mm piece to form the bottom half of the chamber. Then the chamber with the chip was covered with precut silicone rubber gasket sheet and acrylic sheet to form a convenient reusable seal. The entire assembly can be sealed by cutting through-holes in the acrylic sheets and using nuts and bolts or, more simply, with binder clips. The chamber design allows for direct imaging during crystallization without having to open the chamber. Additional information about the storage chamber can be found in the supporting information.

### 2.2. Rapid test chip fabrication with commercial thin-film windows (layer 3)

For testing and screening conditions, it is not necessary to fabricate ultra-thin COC window films. Moreover, to improve vacuum compatibility, Kapton FN or Mylar is preferred over COC. Commercially available COC, Mylar, Kapton, propyl­ene *etc*. are inexpensive as thicker premade films from sources such as Chemplex Industries Inc., Malvern Panalytical and Cole-Parmer (previously SPEX). Kapton films that are 12.5 or 25 µm in thickness are relatively stiff and much easier to handle than 5 µm films (or thinner) used for diffraction measurements. Scalpels or sharp scissors can be used to cut windows [Fig. 1 ▸(*a*), layer 3]. Mylar and polypropyl­ene films are much more flexible. We have found that taping the thin film to a smooth surface is an easy method to remove wrinkles over large areas. Laser-cut acrylic frames [Fig. 1 ▸(*a*), layer 2] can then be adhered to the film to create a half-chip. Following this, users should cut off extra thin film with a scalpel, attach a laser-cut spacer [Fig. 1 ▸(*a*), layer 4], puncture inlet holes and attach the other half-chip. This method is excellent for fabricating chips with more-flexible thin films such as Mylar or propyl­ene. Further details are provided in the supporting information.

### 2.3. Protein crystallization

Lysozyme and PYP were used as model proteins to demonstrate chip performance with counter-diffusion crystallization. Commercially available lyophilized samples of lysozyme were dissolved in Milli-Q water to produce protein solutions of 50 mg ml^−1^. For four channels on a 1″ × 1″ chip with 150 µm spacer, 50 µl of protein solution was sufficient. Two different precipitation buffers were used: 2 *M* NaCl with 0.1 *M* sodium acetate buffer pH 4.6, which yields ∼30 µm crystals, and 3%(*w*/*v*) NaCl in 0.5 *M* Tris–HCl pH 8.5, which yields >70 µm crystals *in situ* without counter diffusion at room temperature. The large lysozyme crystal conditions were adapted from the work of Yu *et al.* (2015 ▸). The results will compare these microbatch conditions with counter diffusion. All samples were sealed in acrylic storage chambers and allowed to grow for 24 h undisturbed.

Purified PYP was concentrated to 100 mg ml^−1^ in the final buffer containing 10 m*M* HEPES, 50 m*M* NaCl pH 7.5. The crystallization buffer used was 100 m*M* MES buffer pH 6.5, 40% PEG 4000 at room temperature. Inside the channels of the chip, the protein-to-buffer ratio was 50:50. The protein solution and buffer were mixed with a pipette a few times before loading. There was 100% buffer inside for counter diffusion. Pure protein solution in the chip during counter diffusion was less effective for PYP crystallization.

### 2.4. Image analysis

**Figure 2:**
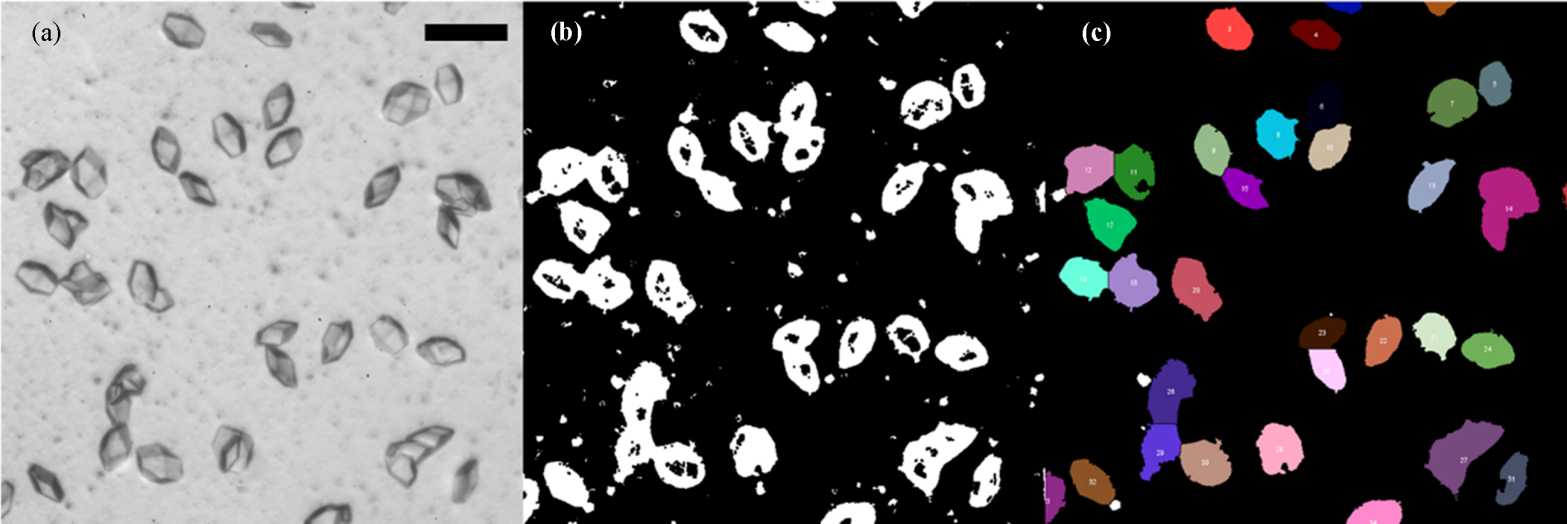
An example image-analysis workflow for the crystals used in this article. (*a*) The original crystal image was (*b*) fed into *Trainable Weka Segmentation* and (*c*) further processed with the extended particle analyzer and watershed features within *BioVoxxel*. The crystals were counted with the default particle analyzer built into *Fiji*. Each color represents a different crystal. The scale bar represents 100 µm.

Here, we first describe the crystal size analysis and then present methods for setting up counter-diffusion crystallization. For each of the four sample channels on one chip [Fig. 1 ▸(*a*)], at least five images were taken, covering ∼80% of the chip. Crystal size and density were obtained using the *Fiji* software (Schindelin *et al.*, 2012 ▸). When the crystal count was very low (<15 per frame), the polygon tool from *Fiji* was used to manually highlight the area of the crystal and the crystal area was directly determined. For higher crystal densities [Fig. 2 ▸(*a*)], *Trainable Weka Segmentation* (Arganda-Carreras *et al.*, 2017 ▸) was utilized to recognize the transparent lysozyme crystals and outline each crystal. The result was fed to the *BioVoxxel* plugin (Brocher, 2022 ▸) to render the area covered by the crystals according to the outline using the extended particle analyzer, to segregate crystal clusters with the watershed tool and to correctly generate a mask labeling the area of each crystal. Afterwards, the default particle analyzer in *Fiji* was used to calculate the area of each crystal and crystal count per frame. An example image workflow is shown in Fig. 2 ▸.

### 2.5. Sample loading and different counter-diffusion designs

**Figure 3:**
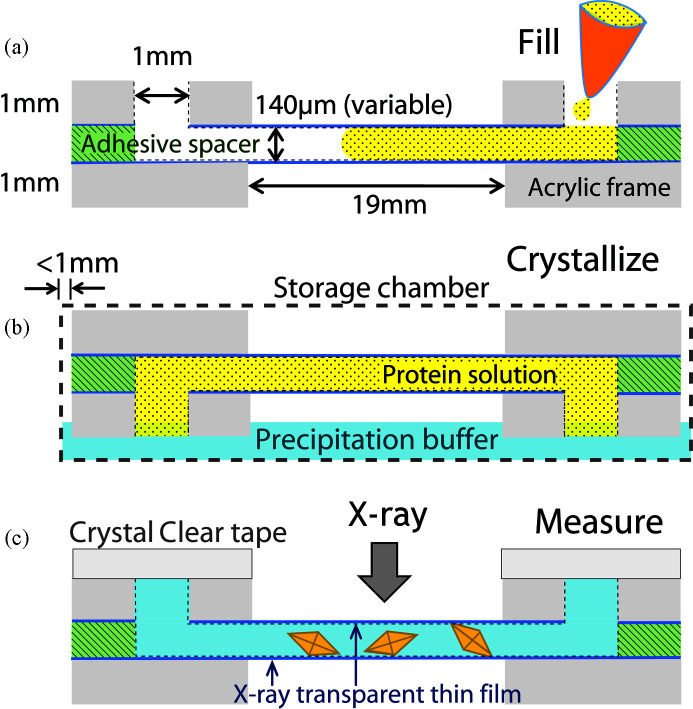
A schematic drawing of a typical counter-diffusion setup with a 1″ × 1″ microfluidic chip. The figures are exaggerated near the inlets compared with the real dimensions. For (*a*) sample loading, the protein solution (yellow) is loaded into the microfluidic channel using a pipette, the adhesive spacer (green) creates the sample flow layer in between the two pieces of thin film (dark blue) and the acrylic frame (gray) prevents the thin film from collapsing. For (*b*) crystallization, after loading, the chip is rotated upside down with open inlets contacting the precipitating buffer (light blue), and the setup is contained in a storage chamber to prevent dehydration. (*c*) When the crystals are fully grown, the precipitating buffer is removed and the chip is sealed with Crystal Clear tape. Before exposure to X-rays, all tape above the thin-film windows is removed, leaving only inlets sealed to prevent dehydration during data collection.

For *in situ* batch crystallization on the chips, the microfluidic channel was typically filled with a 50:50 mixture of protein solution and precipitation buffer. For counter diffusion, only protein solution was loaded in the microfluidic channel. Importantly, this effectively doubles the amount of protein in the microfluidic channel for forming crystals. For counter diffusion, precipitation buffer was added to a reservoir space. The protein-loaded chip was put into the chamber contacting the precipitation buffer. A schematic drawing of the process is shown in Fig. 3 ▸. The sample-loading inlets/outlets are left open to allow diffusion between the protein solution and precipitation buffer. Due to the microchannel geometry of the chip (a high aspect ratio of channel length to thickness), counter-diffusion-dominated mass transfer will occur with little convection in the system. The dimensionless Grashof number, Gr, describes the contribution of convective mass transfer (buoyancy to viscous drag forces) in the counter-diffusion system as
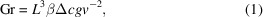
where *L* is the characteristic length, β is the solution expansion coefficient due to concentration change, Δ*c* is the concentration change, *g* is the gravitational constant on Earth and *v* is the solution kinematic viscosity (Otálora *et al.*, 2009 ▸). By tuning the characteristic length alone (chip channel thickness and use of hydro­gels), the Grashof number and the convection flow in the system can be greatly reduced.

Next, we describe some of the chip counter-diffusion setups that can be easily implemented for on-chip crystallization. These designs can be adapted to different user needs and inspire new designs. The reduction factor in the Grashof number for each design compared with a 3 mm hanging-drop crystallization (Gr_0_) is also shown in Table 1 ▸. Furthermore, one can easily exchange the precipitation buffer during crystallization to achieve maximum tunability of the crystal density and size.

**Figure 4:**
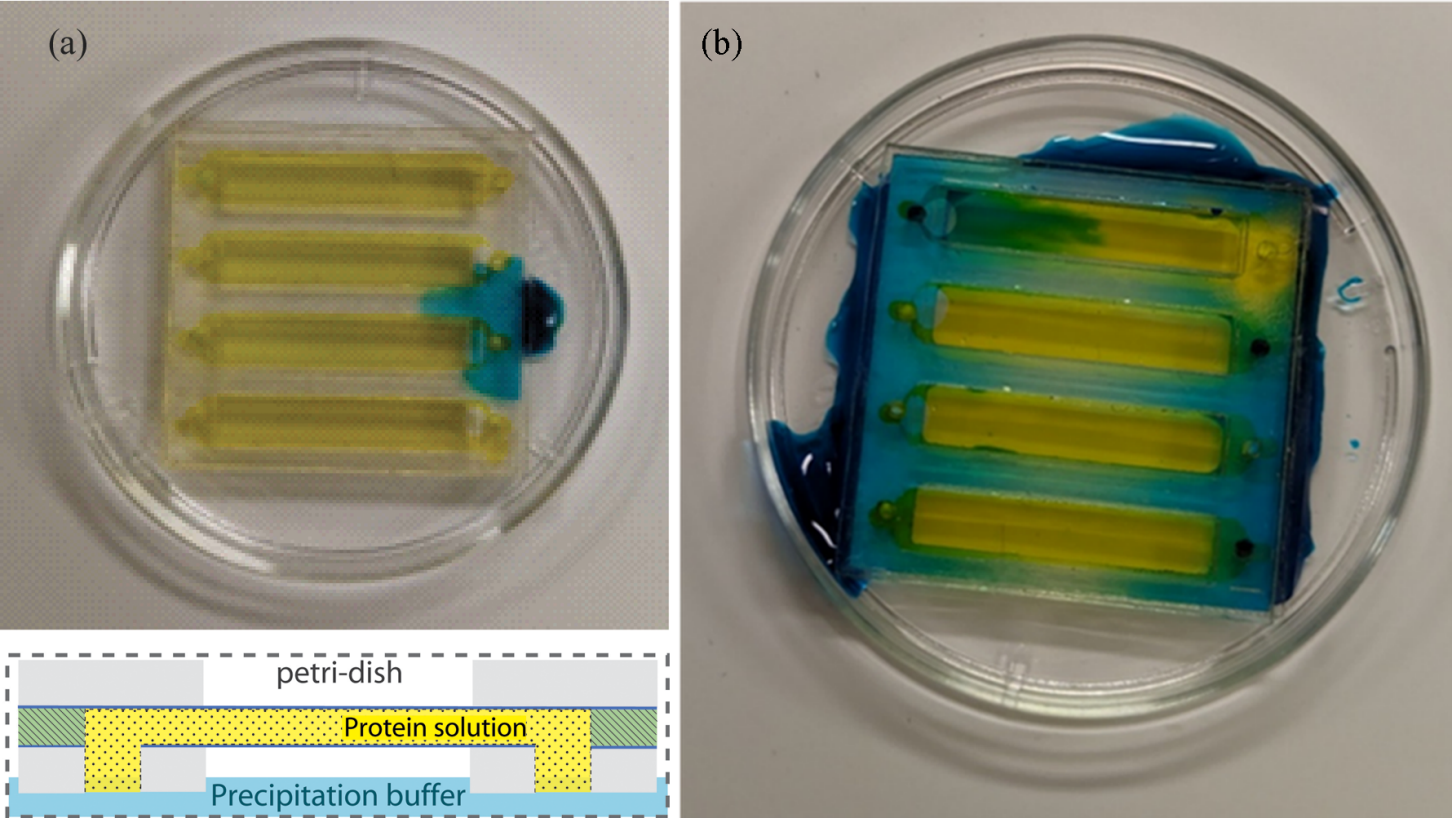
Photographs and a schematic drawing of the ‘upside down in dish’ setup. The yellow dye visualizes the protein solution. After sample loading, the chip was placed upside down in the petri dish and the precipitation buffer (blue dye) was added at the bottom of the petri dish at the edge of the chip. (*a*) Initial deposition of the precipitation buffer. (*b*) After adding sufficient precipitation buffer, the solution should spread underneath the acrylic frame without entering the microfluidic channel. However, petri dishes are less reliable compared with customized storage chambers and can leak if there is extra stress between the petri-dish wall and the corner of the chip. The top channel shows a case where the precipitation buffer flowed into the protein channel resulting in batch crystallization conditions.

Options for on-chip counter-diffusion crystallization from easy to difficult fabrication include:

(1) Upside down in a petri dish with parafilm (Fig. 4 ▸). This easy setup uses an upside-down 35 mm petri dish and a standard microfluidic chip for counter diffusion. It is ideal for initial testing of crystallization conditions. The chip windows can be made of thicker inexpensive premade commercial films. Such chips are quicker to fabricate but have significantly more background scatter than chips with thin-film windows. To start crystallization, the chip is loaded with protein solution and placed inlet side down in the petri dish. Afterwards, precipitation buffer is added to the bottom of the petri dish.

(2) Hydro­gel reservoir chips (Fig. S1 of the supporting information). Hydro­gels such as agarose or pluronic F127 can be used to improve crystal quality [*i.e.* decrease *L* in equation (1)], fix the crystals in place during measurements or accommodate special crystallization conditions. In this case, wells are cut in the top (the side with inlet holes) PMMA frame (layer 2) for each of the sample channels. Thus, each sample channel has a separate reservoir. After the sample has been loaded, one inlet should be sealed with nail polish to prevent convection flow during handling. The reservoir is then consecutively filled with the hydro­gel and with precipitation buffer. Only one inlet hole is needed as the sample channel will connect to the reservoir at the edge of the spacer layer. Fig. S1 provides detailed images of the setup. The separated channels and reservoirs allow different protein solutions and precipitation buffer conditions to be tested on the same chip.

(3) Reservoir in chamber – direct (Fig. S2). This setup allows direct contact between the protein solution and the precipitation buffer, and is suitable for more-viscous buffers and protein solutions. For viscous samples, convection flow is already low, so no extra counter-diffusion barrier is needed. Here, the buffer reservoirs are built separately into the reusable acrylic chambers for sample storage. When the chip is inserted into the storage chamber, the inlets are directly above these precipitation buffer reservoirs. Each channel with two contacting reservoirs on either end is independent, and the buffer can be switched out at any time during crystallization or storage. Although easy to fabricate, this type of chamber may leak between buffer reservoirs if they are overfilled. Conversely, if the chip and/or reservoirs are underfilled, contact and diffusion between the two may not occur.

(4) Reservoir in chamber – filter paper (Fig. S3). This design also has separated channels and built-in reservoirs within the storage chambers. Each buffer reservoir is connected to the corresponding inlet by a small piece of filter paper. This setup allows steady diffusion of the precipitation buffer and is suitable for low-viscosity solutions or when there are large viscosity differences between the buffer and protein solution. The filter paper directs and contains the precipitation buffer to minimize any cross-contamination between the channels. The setup can be further secured by covering the entire acrylic chamber panel with a piece of Crystal Clear tape, sealing in all reservoirs and filter-paper pieces.

General recommendations include:

(i) For easy loading of very viscous solutions into the microfluidic channel, load at one end of the channel by placing a droplet above the inlet and sucking (slight vacuum) from the other side of the channel. This is easily accomplished by in­ser­ting a compressed 1 ml pipette into the other inlet/outlet chan­nel and slowly releasing the pipette until all the viscous liquid is drawn into the channel. Pipette tips for loading any of the solutions can also be cut to better fit to the 1 mm-diameter inlet.

(ii) Hydro­gel options such as agarose and pluronic gel are compatible with all designs. Mixing with the protein solution and sample loading should be carried out before gelling. Incorporating hydro­gels in channels can improve crystal quality, can prevent dehydration and movement in the chip during measurements, and is especially helpful for measurements under vacuum. However, using unsealed hydro­gels as diffusion barriers in storage chambers is not recommended because they tend to fog up the petri dish or acrylic storage chamber due to high water content, which can interfere with observation and imaging during crystallization.

(iii) Covering chip inlets with dialysis membranes cut from dialysis tubes is generally not advisable. No significant differences in nucleation and growth of crystals between setups with and without dialysis membranes have been observed. Furthermore, ensuring a proper seal is challenging, adding unnecessary complications to the design.

### 2.6. X-ray diffraction measurements

**Figure 5:**
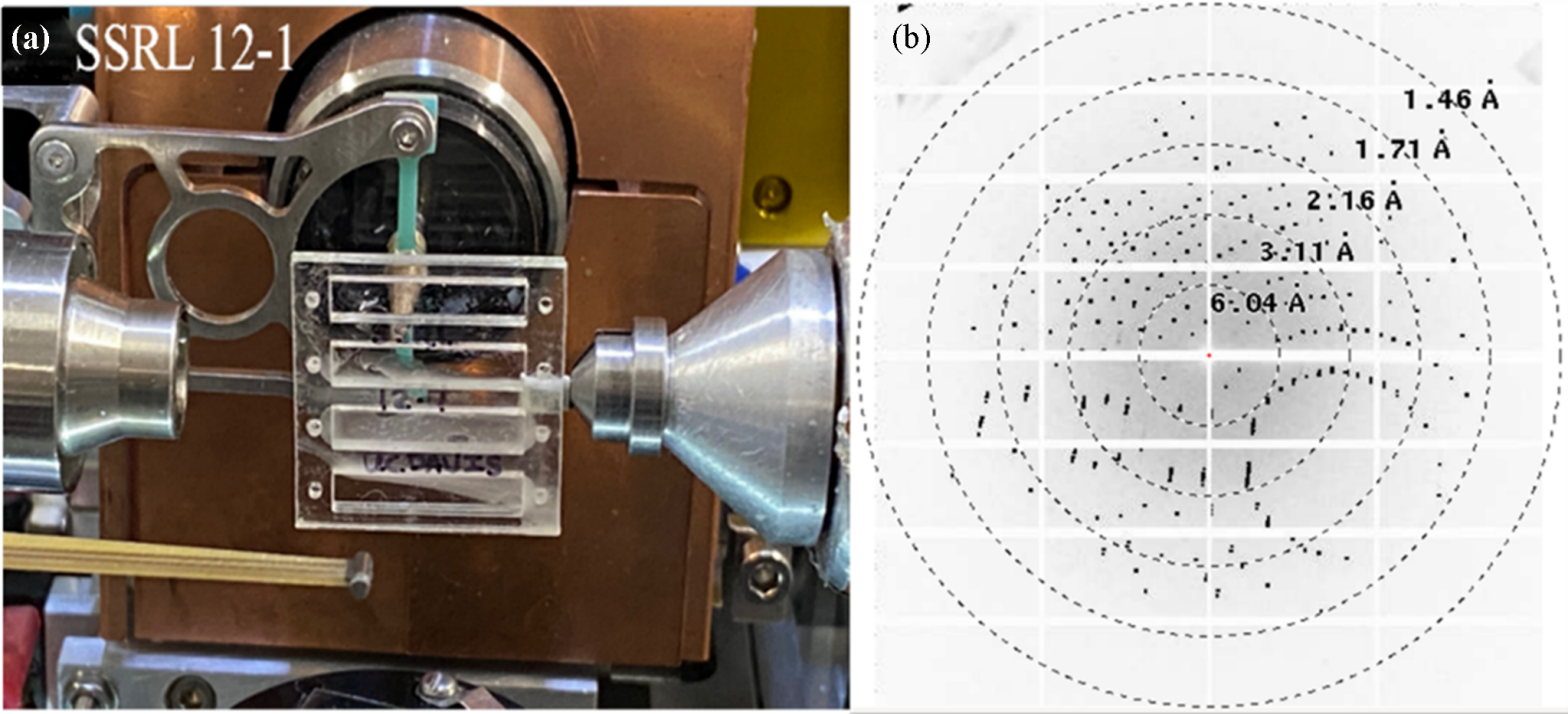
(*a*) A chip mounted at the 12-1 beamline at SSRL. (*b*) A diffraction image of PYP taken at SSRL 12-1 from the crystal shown in Fig. 7.

PYP was crystallized within an upside-down COC chip in a petri dish, which was sealed with parafilm; the simplest counter-diffusion setup. Crystals grown in the chip were measured at ambient temperature at beamline 12-1 at the Stanford Synchrotron Radiation Lightsource (SSRL). Shortly before mounting, the storage chamber was opened and the Crystal Clear tape protecting the chip windows was removed (the inlets were still sealed with tape to prevent dehydration during the measurements). As the chips are wider than a standard magnetic mount, the chip was attached by its edge to a thin piece of acrylic (0.5 mm), which was then secured onto a slotted holder with a magnetic base using a screw. This assembly was subsequently mounted onto the goniometer at the beamline. Diffraction data were collected at a wavelength of 0.9799 Å, with a beam size of 0.005 × 0.04 mm, using an Eiger X 16M detector (Dectris AG) at a detector distance of 0.2 m. The beamline’s sample-holder translational motors were used to align and center individual single crystals in the beam path, using the inline high-resolution camera to identify each crystal. Datasets were collected from these centered single crystals in 30° rotations. Diffraction data from nine sweeps were merged to complete datasets for PYP with *xia2* (Winter, 2010 ▸; Winter *et al.*, 2018 ▸; Beilsten-Edmands *et al.*, 2020 ▸; Agirre *et al.*, 2023 ▸), and the structure was refined with *Phenix 1.19* (Liebschner *et al.*, 2019 ▸) using the *P*6_5_ crystal form (Van Aalten *et al.*, 2000 ▸) as a starting model. An example diffraction pattern of a PYP crystal grown by counter diffusion is shown in Fig. 5 ▸.

## 3. Results and discussion

### 3.1. Images of crystals obtained through counter diffusion

**Figure 6:**
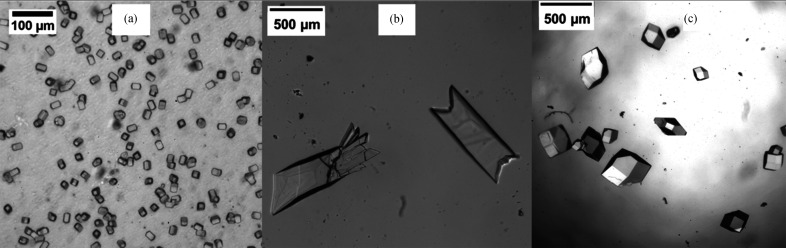
Lysozyme crystals obtained *in situ* on chip through (*a*) microbatch crystallization in a 48 µm channel, (*b*) counter diffusion in a 48 µm channel and (*c*) counter diffusion in a 140 µm channel. The thinner 48 µm channel resulted in a flattened crystal morphology. Tuning of the channel layer thickness is simply accomplished by selection of the adhesive layer thickness [layer 4, Fig. 1 ▸(*a*)].

**Figure 7:**
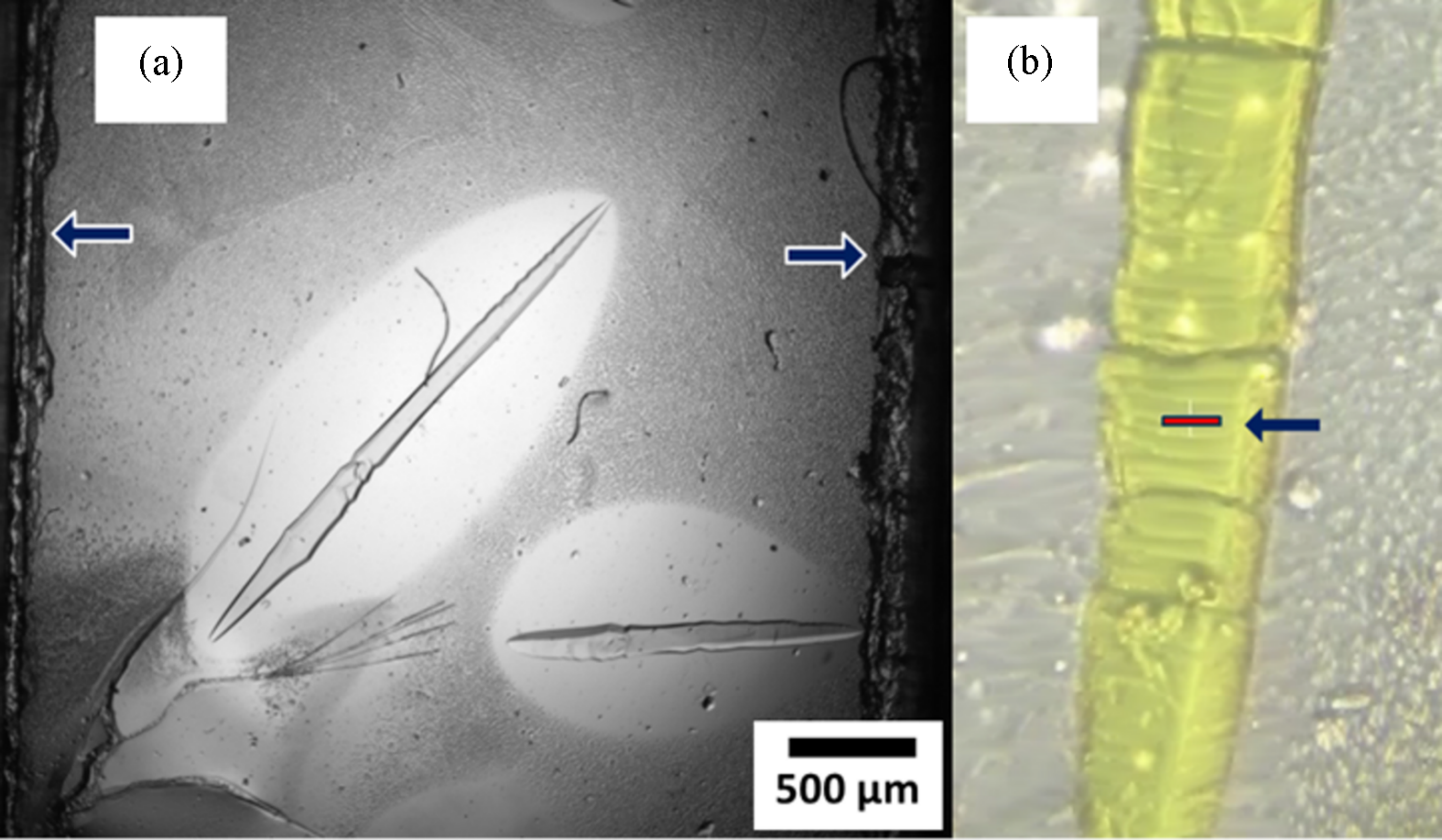
(*a*) PYP crystals obtained on chip by counter diffusion using the simplest ‘upside-down chip in petri dish’ setup. The arrows indicate the channel width (3 mm) and sides of the adhesive spacer. In the lower left corner, the PYP crystal damaged the 2.7 µm-thick COC window. The lighter area around the crystal is due to a dramatic reduction in protein concentration, demonstrating that the crystals are growing in the absence of convection. (*b*) An image of the PYP crystal mounted at the SSRL 12-1 beamline during diffraction measurements. The diffraction image in Fig. 5 ▸ was from this crystal. The bar highlighted by the arrow indicates the beam size of 5 × 40 µm.

The crystals depicted in Fig. 6 ▸ were grown using traditional microbatch conditions and the simplest ‘upside-down chip in petri dish’ setup (Fig. 4 ▸). The counter-diffusion-grown crystals were significantly larger (>100 µm) than those obtained with the microbatch method (∼30 µm). Additionally, there were morphological differences between the crystals. The counter-diffusion crystals grew to span the thickness of the standard 48 µm channel [the thickness of the adhesive spacer, layer 4 in Fig. 1 ▸(*a*)] and appear flattened, as shown in Fig. 6 ▸(*b*). The microchannel/sample layer thickness is simply controlled by the thickness of the adhesive-spacer layer. Increasing the thickness of the adhesive-spacer layer to 150 µm resulted in large more-faceted lysozyme crystals [Fig. 6 ▸(*c*)]. PYP crystals, shown in Fig. 7 ▸, also grew to span the spacer layer thickness. Diffusion-dominated crystallization is clearly visible by the difference in bright-light imaging. A light, bright, depleted region of low protein concentration is clearly discernable around the PYP crystals. Despite a crack in the chip window film [lower left corner in Fig. 7 ▸(*a*)], the crystal remained intact and hydrated in the storage chamber. After crystal growth, the chips were removed from the counter-diffusion setup, sealed with Crystal Clear tape [Fig. 1 ▸(*a*), layer 1] and placed in a storage chamber. The storage chambers are almost identical to the counter-diffusion chambers (Gu *et al.*, 2023 ▸) but without the precipitation buffer or reservoirs. Storage chambers minimize the free volume that the chip sample is equilibrating with. They are highly effective in preserving the crystals from damage and dehydration. Storage-chamber performance and additional fabrication details are described by Gu *et al.* (2023 ▸). Chips fabricated with commercially available Mylar films gave a very similar loading and storage stability result (Fig. S4). As an aside, we recently tested the storage chambers with photosystem I (PSI) crystals, which are extraordinarily sensitive to dehydration (Fromme & Witt, 1998 ▸). After loading PSI crystal slurry into a chip and placing the chip in a sample chamber, PSI crystals survived in the chip under ambient conditions for two weeks (Fig. S8).

### 3.2. Nucleation and growth

**Figure 8:**
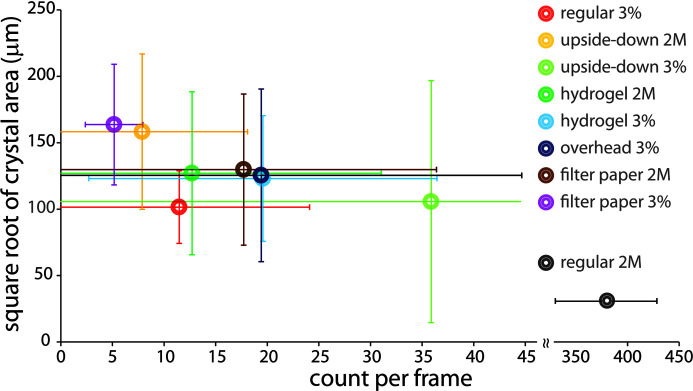
Crystal size and density obtained using different crystallization conditions and counter-diffusion setups. The error bars represent the variation in crystal size and the number of crystals per microscopy image from the three independent trials. At least four image frames were taken over different regions of the chip to account for variation in crystal density (counts per frame). Different crystallization conditions were used: ‘2M’ stands for 2 *M* NaCl with 0.1 *M* sodium acetate buffer pH 4.6, which yields regular sized (∼30 µm) crystals in microbatch crystallization; ‘3%’ stands for 3%(*w*/*v*) NaCl in 0.5 *M* Tris–HCl pH 8.5, conditions that typically yield larger (∼80 µm) crystals in microbatch crystallization; and ‘regular’ stands for typical microbatch conditions in a microfluidic chip. These microbatch conditions were achieved with a 1:1 mixture of protein solution and precipitating buffer in the channels. Counter-diffusion setups included ‘upside down’ for ‘upside-down chip in chamber’, where the chip was placed upside down in a petri dish and contacted with a reservoir of precipitation buffer; ‘hydro­gel’ for a 30% pluronic F127 hydro­gel reservoir chip; and ‘overhead’ and ‘filter paper’ for a reservoir in a storage chamber with direct contact with the precipitation buffer or indirect contact through filter paper, respectively.

Counter-diffusion setups consistently produced larger crystals (>100 µm) compared with microbatch setups (∼30 µm) with identical chip dimensions (Fig. 8 ▸). Typically, larger crystals were more sparsely distributed within the channels. Crystals grown through counter diffusion exhibited comparable or even superior results to those achieved by specifically choosing the buffer conditions that yielded larger crystals in microbatch setups [such as 3%(*w*/*v*) NaCl in 0.5 *M* Tris–HCl pH 8.5 for lysozyme]. The variation in crystal sizes obtained from different designs was not statistically significant.

The simplicity and flexibility of the polymer microfluidic chips and counter-diffusion setups are a significant advancement. Users can select the most suitable design for their specific needs. One can combine precipitation buffer conditions and counter-diffusion techniques to cultivate larger crystals. For preliminary experiments, it is recommended to start with the simple ‘upside-down in dish’ configuration, which does not require altering the chip design or manufacturing a chamber. As experiments progress to more-complex scenarios, such as challenging crystallization conditions, pushing the limits for even larger crystal sizes or progressive changes in crystallization conditions by exchanging the precipitation buffer during crystallization, the choice of design should be guided by a thorough evaluation of the pros and cons outlined in Table 1 ▸.

While the trials clearly showed that counter diffusion directly affects the nucleation rates and growth rates, resulting in substantially fewer and larger protein crystals, there were some limitations. Images used to generate the crystal size and density data were randomly selected across the microfluidic channels. However, the crystal size and density varied even with the same conditions. Additionally, frequent imaging and manipulation of the crystallization setups during the first 6 h of crystallization had a great impact on the crystal nucleation rates and density. Handling resulted in some convection and smaller more-numerous crystals compared with setups that were left for at least a day to grow. It is therefore recommended to not move or handle the counter-diffusion setup during the typical crystallization time to maximize the benefits from the low mass transportation rates.

### 3.3. X-ray diffraction measurements

**Figure 9:**
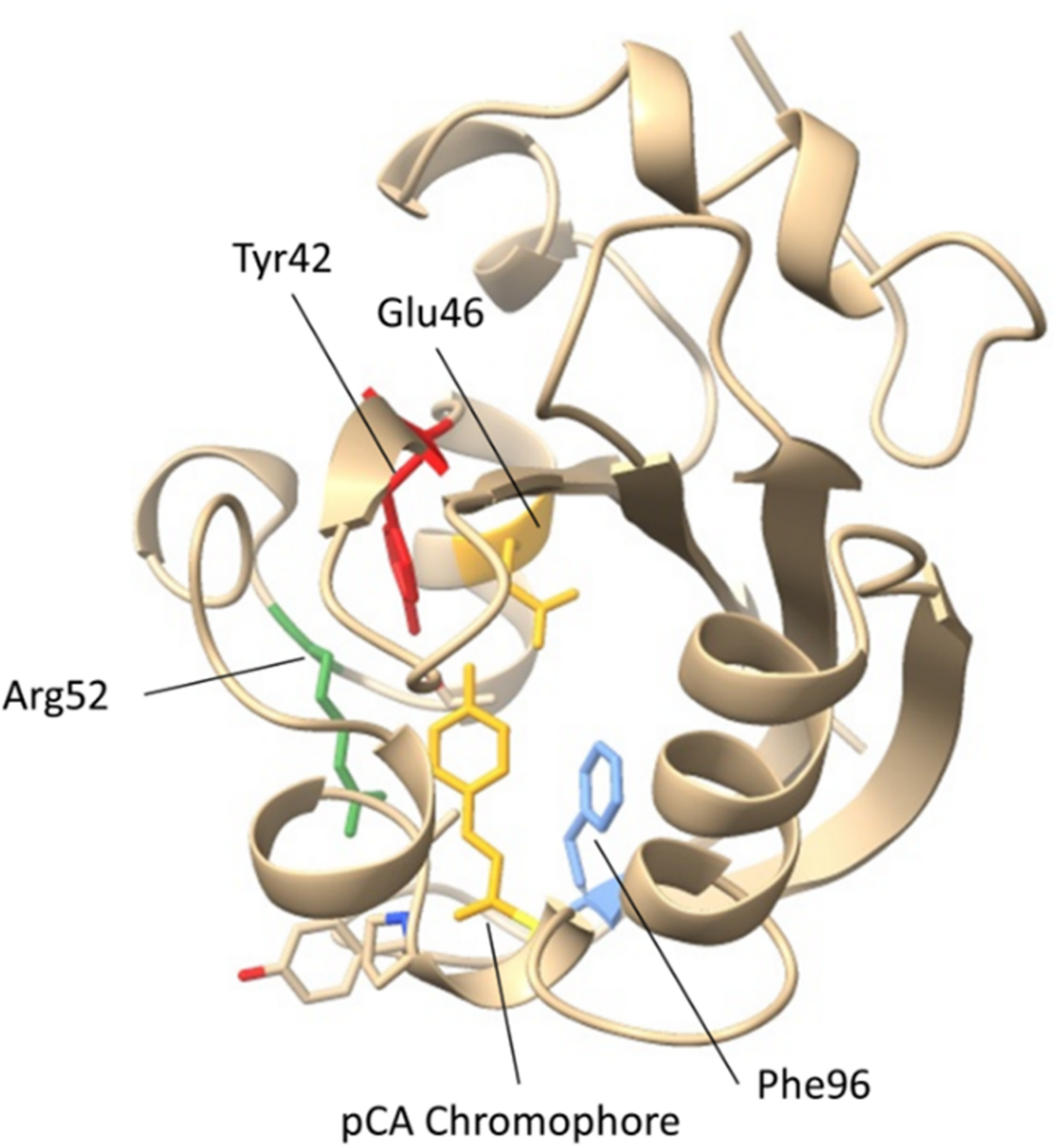
The refined structure of PYP collected at beamline 12-1 at SSRL. The p-coumaric acid chromophore (pCA), Tyr42, Glu46, Arg52 and Phe96 are shown.

The simplest setup of an upside-down chip in a petri dish was used to grow extra-large PYP crystals within two days of scheduled beam time at SSRL. The use of counter diffusion within the chip (*in situ*) greatly facilitated efficient data collection. The large PYP crystals were easy to visualize, focus on and center with the goniometer. Seventeen 30° rotation wedges were collected from three crystals within 40 min, achieving high-resolution diffraction to 1.32 Å. Data from nine sweeps were merged to form complete datasets for the measured proteins, resulting in a high completeness of 99.36%. The high-resolution diffraction datasets from PYP were refined with excellent merging statistics, yielding *R*_work_/*R*_free_ values of 0.170/0.177. The PYP structure obtained was comparable to previously reported results, but with higher resolution (Pandey *et al.*, 2020 ▸; Tenboer *et al.*, 2014 ▸). Detailed data-collection and refinement statistics, demonstrating this improvement, are presented in Table 2 ▸. The refined structure is shown in Fig. 9 ▸ and the PYP structure was deposited with PDB ID 9cgh.

## 4. Conclusions

Straightforward and effective counter diffusion for growing large crystals within fixed-target microfluidic chips appropriate for *in situ* diffraction was demonstrated. Multiple designs of counter-diffusion setups and compatible storage chambers that are effective, inexpensive and highly customizable were described to allow users to tune nucleation and crystal growth in order to optimize crystal quality and size under different sample conditions. High-quality protein crystals grown directly within the low X-ray background chip minimize sample consumption and enable immediate X-ray diffraction measurements without additional crystal handling. Experimental results with lysozyme and photoactive yellow protein validated the chip’s performance, demonstrating its potential to yield high-quality diffraction data.

Overall, this work is a valuable tool for structural biologists, facilitating more efficient and precise protein crystallography experiments. The microfluidic fixed-target chip stands as a promising innovation in the field, poised to enhance the capabilities of X-ray crystallography and improve the structural analysis of proteins.

## Supplementary Material

Supporting information. DOI: 10.1107/S1600576724007544/jo5110sup1.pdf

PDB reference: Photoactive yellow protein crystallized *in situ* on a cyclic olefin copolymer microfluidic chip through counter diffusion, 9cgh

## Figures and Tables

**Table 1:** Comparison of counter-diffusion setup designs and reduction factor in Grashof number [Gr, equation (1)] for a 150 µm channel height in a 1″ × 1″ chip relative to a 3 mm hanging droplet (Gr_0_)

Design	Pros	Cons	Reduction factor (Gr_0_/Gr) [equation (1)]
Upside-down chip in petri dish with parafilm seal around dish (Fig. 4 ▸)	Quick, easy and flexible setup; best for batch experiments.	Less reliable due to leakage; may be difficult to image in the petri dish.	∼10^4^
Upside-down chip in chamber (Fig. 3 ▸)	Straightforward setup with long-term storage; easy to transport.	Cross-contamination between channels; cannot vary buffer conditions in a single setup.	∼10^4^
Hydro­gel reservoir chip (Fig. S1)	Agarose, pluronic or another gel as barrier; separated channels for different buffer conditions.	More difficult to fabricate and seal.	∼10^12^ (assuming 200 nm pore size)
Reservoir in chamber – direct (Fig. S2)	Good for more-viscous buffer and protein solution. Easy to fabricate.	May leak between buffer reservoirs if they are overfilled and not produce crystals when underfilled.	∼10^4^
Reservoir in chamber – filter paper (Fig. S3)	Good for lower-viscosity solutions or large viscosity differences.	More difficult to set up.	∼3 × 10^8^ (assuming 10 µm pore size)

**Table 2:** Data-collection and refinement statistics of PYP Values in parentheses are for the highest resolution shell.

Wavelength (Å)	0.97946
Resolution range (Å)	35.68–1.32 (1.35–1.32)
Space group	*P*6_5_
Unit-cell parameters (Å, °)	41.194, 41.194, 117.819, 90, 90, 120
Unique reflections	26336 (1771)
Multiplicity	13.83 (5.75)
Completeness (%)	99.36 (94.10)
〈*I*/σ(*I*)〉	22.98 (1.52)
Wilson *B* factor	16.93
*R*_merge_	0.063 (1.173)
*R*_meas_	0.065 (1.282)
*R*_p.i.m._	0.016 (0.495)
CC_1/2_	0.9995 (0.4369)
Reflections used in refinement	26336 (1771)
Reflections used for *R*_free_	2013 (138)
*R*_work_	0.1704 (0.2331)
*R*_free_	0.1772 (0.2709)
Number of non-hydrogen atoms	1108
Macromolecules	1025
Ligands	11
Solvent	72
Protein residues	124
RMS (bonds)	0.007
RMS (angles)	0.96
Ramachandran favored (%)	96.72
Ramachandran allowed (%)	3.28
Ramachandran outliers (%)	0
Rotamer outliers (%)	3.33
Clashscore	4.31
Average *B* factor	23.22
Macromolecules	22.7
Ligands	14.32
Solvent	31.98

## References

[bb39] Agirre, J., Atanasova, M., Bagdonas, H., Ballard, C. B., Baslé, A., Beilsten-Edmands, J., Borges, R. J., Brown, D. G., Burgos-Mármol, J. J., Berrisford, J. M., Bond, P. S., Caballero, I., Catapano, L., Chojnowski, G., Cook, A. G., Cowtan, K. D., Croll, T. I., Debreczeni, J. É., Devenish, N. E., Dodson, E. J., Drevon, T. R., Emsley, P., Evans, G., Evans, P. R., Fando, M., Foadi, J., Fuentes-Montero, L., Garman, E. F., Gerstel, M., Gildea, R. J., Hatti, K., Hekkelman, M. L., Heuser, P., Hoh, S. W., Hough, M. A., Jenkins, H. T., Jiménez, E., Joosten, R. P., Keegan, R. M., Keep, N., Krissinel, E. B., Kolenko, P., Kovalevskiy, O., Lamzin, V. S., Lawson, D. M., Lebedev, A. A., Leslie, A. G. W., Lohkamp, B., Long, F., Malý, M., McCoy, A. J., McNicholas, S. J., Medina, A., Millán, C., Murray, J. W., Murshudov, G. N., Nicholls, R. A., Noble, M. E. M., Oeffner, R., Pannu, N. S., Parkhurst, J. M., Pearce, N., Pereira, J., Perrakis, A., Powell, H. R., Read, R. J., Rigden, D. J., Rochira, W., Sammito, M., Sánchez Rodríguez, F., Sheldrick, G. M., Shelley, K. L., Simkovic, F., Simpkin, A. J., Skubak, P., Sobolev, E., Steiner, R. A., Stevenson, K., Tews, I., Thomas, J. M. H., Thorn, A., Valls, J. T., Uski, V., Usón, I., Vagin, A., Velankar, S., Vollmar, M., Walden, H., Waterman, D., Wilson, K. S., Winn, M. D., Winter, G., Wojdyr, M. & Yamashita, K. (2023). *Acta Cryst.* D**79**, 449–461.

[bb1] Aquila, A., Hunter, M. S., Doak, R. B., Kirian, R. A., Fromme, P., White, T. A., Andreasson, J., Arnlund, D., Bajt, S., Barends, T. R. M., Barthelmess, M., Bogan, M. J., Bostedt, C., Bottin, H., Bozek, J. D., Caleman, C., Coppola, N., Davidsson, J., DePonte, D. P., Elser, V., Epp, S. W., Erk, B., Fleckenstein, H., Foucar, L., Frank, M., Fromme, R., Graafsma, H., Grotjohann, I., Gumprecht, L., Hajdu, J., Hampton, C. Y., Hartmann, A., Hartmann, R., Hau-Riege, S., Hauser, G., Hirsemann, H., Holl, P., Holton, J. M., Hömke, A., Johansson, L., Kimmel, N., Kassemeyer, S., Krasniqi, F., Kühnel, K., Liang, M., Lomb, L., Malmerberg, E., Marchesini, S., Martin, A. V., Maia, F. R. N. C., Messerschmidt, M., Nass, K., Reich, C., Neutze, R., Rolles, D., Rudek, B., Rudenko, A., Schlichting, I., Schmidt, C., Schmidt, K. E., Schulz, J., Seibert, M. M., Shoeman, R. L., Sierra, R., Soltau, H., Starodub, D., Stellato, F., Stern, S., Strüder, L., Timneanu, N., Ullrich, J., Wang, X., Williams, G. J., Weidenspointner, G., Weierstall, U., Wunderer, C., Barty, A., Spence, J. C. H. & Chapman, H. N. (2012). *Opt. Express*, **20**, 2706–2716.

[bb2] Arganda-Carreras, I., Kaynig, V., Rueden, C., Eliceiri, K. W., Schindelin, J., Cardona, A. & Sebastian Seung, H. (2017). *Bioinformatics*, **33**, 2424–2426. 10.1093/bioinformatics/btx180 28369169

[bb3] Beilsten-Edmands, J., Winter, G., Gildea, R., Parkhurst, J., Waterman, D. & Evans, G. (2020). *Acta Cryst.* D**76**, 385–399. 10.1107/S2059798320003198 PMC7137103 32254063

[bb4] Borgstahl, G. E., Williams, D. R. & Getzoff, E. D. (1995). *Biochemistry*, **34**, 6278–6287. 10.1021/bi00019a004 7756254

[bb5] Brocher, J. (2022). *biovoxxel/BioVoxxel-Toolbox: BioVoxxel Toolbox* (v2.5.3), https://zenodo.org/records/5986130.

[bb6] Clegg, W. (2019). *Philos. Trans. R. Soc. A*, **377**, 20180239. 10.1098/rsta.2018.0239 PMC6501891 31030659

[bb7] Dhouib, K., Khan Malek, C., Pfleging, W., Gauthier-Manuel, B., Duffait, R., Thuillier, G., Ferrigno, R., Jacquamet, L., Ohana, J., Ferrer, J. L., Théobald-Dietrich, A., Giegé, R., Lorber, B. & Sauter, C. (2009). *Lab Chip*, **9**, 1412–1421. 10.1039/b819362b 19417908

[bb8] Doak, R. B., Nass Kovacs, G., Gorel, A., Foucar, L., Barends, T. R. M., Grünbein, M. L., Hilpert, M., Kloos, M., Roome, C. M., Shoeman, R. L., Stricker, M., Tono, K., You, D., Ueda, K., Sherrell, D. A., Owen, R. L. & Schlichting, I. (2018). *Acta Cryst.* D**74**, 1000–1007. 10.1107/S2059798318011634 PMC6173051 30289410

[bb9] Fromme, P. & Witt, H. T. (1998). *Biochim. Biophys. Acta*, **1365**, 175–184.

[bb10] García-Ruiz, J. M. (2003). *Methods Enzymol.* **368**, 130–154. 10.1016/S0076-6879(03)68008-0 14674272

[bb11] Gavira, J. A., Otálora, F., González-Ramírez, L. A., Melero, E., Driessche, A. E. V. & García-Ruíz, J. M. (2020). *Crystals*, **10**, 68.

[bb12] Gilbile, D., Shelby, M. L., Lyubimov, A. Y., Wierman, J. L., Monteiro, D. C., Cohen, A. E., Russi, S., Coleman, M. A., Frank, M. & Kuhl, T. L. (2021). *Lab Chip*, **21**, 4831–4845. 10.1039/d1lc00810b PMC8915944 34821226

[bb13] González-Ramírez, L. A., Ruiz-Martínez, C. R., Estremera-Andújar, R. A., Nieves-Marrero, C. A., García-Caballero, A., Gavira, J. A., López-Garriga, J. & García-Ruiz, J. M. (2017). *Cryst. Growth Des.* **17**, 6780–6786.

[bb14] Gu, K. K., Liu, Z., Narayanasamy, S. R., Shelby, M. L., Chan, N., Coleman, M. A., Frank, M. & Kuhl, T. L. (2023). *Biomicrofluidics*, **17**, 051302. 10.1063/5.0167164 PMC10576627 37840537

[bb15] Hansen, C. L., Skordalakes, E., Berger, J. M. & Quake, S. R. (2002). *Proc. Natl Acad. Sci. USA*, **99**, 16531–16536. 10.1073/pnas.262485199 PMC139178 12486223

[bb16] Hunter, M. S., Segelke, B., Messerschmidt, M., Williams, G. J., Zatsepin, N. A., Barty, A., Benner, W. H., Carlson, D. B., Coleman, M., Graf, A., Hau-Riege, S. P., Pardini, T., Seibert, M. M., Evans, J., Boutet, S. & Frank, M. (2014). *Sci. Rep.* **4**, 6026. 10.1038/srep06026 PMC4129423 25113598

[bb17] Imamoto, Y., Ito, T., Kataoka, M. & Tokunaga, F. (1995). *FEBS Lett.* **374**, 157–160. 10.1016/0014-5793(95)01096-w 7589524

[bb18] Kober, U. A., Ogbuoji, E. A., Hutchinson, J. A., Mueser, T. C. & Schall, C. A. (2023). *J. Appl. Cryst.* **56**, 1057–1065. 10.1107/S1600576723004958 PMC10405592 37555216

[bb19] Konold, P. E., You, T., Bielecki, J., Valerio, J., Kloos, M., Westphal, D., Bellisario, A., Varma Yenupuri, T., Wollter, A., Koliyadu, J. C. P., Koua, F. H. M., Letrun, R., Round, A., Sato, T., Mészáros, P., Monrroy, L., Mutisya, J., Bódizs, S., Larkiala, T., Nimmrich, A., Alvarez, R., Adams, P., Bean, R., Ekeberg, T., Kirian, R. A., Martin, A. V., Westenhoff, S. & Maia, F. R. N. C. (2023). *IUCrJ*, **10**, 662–670. 10.1107/S2052252523007972 PMC10619454 37721770

[bb20] Kort, R., Hoff, W. D., Van West, M., Kroon, A. R., Hoffer, S. M., Vlieg, K. H., Crielaand, W., Van Beeumen, J. J. & Hellingwerf, K. J. (1996). *EMBO J.* **15**, 3209–3218. PMC451869 8670821

[bb21] Kurz, M., Blattmann, B., Kaech, A., Briand, C., Reardon, P., Ziegler, U. & Gruetter, M. G. (2012). *J. Appl. Cryst.* **45**, 999–1008.

[bb22] Lee, D., Baek, S., Park, J., Lee, K., Kim, J., Lee, S. J., Chung, W. K., Lee, J. L., Cho, Y. & Nam, K. H. (2019). *Sci. Rep.* **9**, 6971. 10.1038/s41598-019-43485-z PMC6502819 31061502

[bb23] Liebschner, D., Afonine, P. V., Baker, M. L., Bunkóczi, G., Chen, V. B., Croll, T. I., Hintze, B., Hung, L.-W., Jain, S., McCoy, A. J., Moriarty, N. W., Oeffner, R. D., Poon, B. K., Prisant, M. G., Read, R. J., Richardson, J. S., Richardson, D. C., Sammito, M. D., Sobolev, O. V., Stockwell, D. H., Terwilliger, T. C., Urzhumtsev, A. G., Videau, L. L., Williams, C. J. & Adams, P. D. (2019). *Acta Cryst.* D**75**, 861–877. 10.1107/S2059798319011471 PMC6778852 31588918

[bb51] Liu, Z., Gu, K. K., Shelby, M. L., Gilbile, D., Lyubimov, A. Y., Russi, S., Cohen, A. E., Narayanasamy, S. R., Botha, S., Kupitz, C., Sierra, R. G., Poitevin, F., Gilardi, A., Lisova, S., Coleman, M. A., Frank, M. & Kuhl, T. L. (2023). *Acta Cryst.* D**79**, 944–952. 10.1107/S2059798323007027 PMC10565732 37747292

[bb50] Maes, D., Crabeel, M., Van de Weerdt, C., Martial, J., Peeters, E., Charlier, D., Decanniere, K., Vanhee, C., Wyns, L. & Zegers, I. (2006). *Acta Cryst.* F**62**, 1294–1297. 10.1107/S1744309106050561 PMC2225363 17142921

[bb24] Malin, L. E., Graves, W. S., Spence, J., Weiss, K., Zhang, C., Li, R. K. & Yang, J. (2018). *Proceedings of the 9th International Particle Accelerator Conference (IPAC2018)*, 29 April–4 May 2018, Vancouver, BC, Canada, pp. 1553–1555. TUPML011. JACoW Publishing.

[bb25] Meyer, T. E. (1985). *Biochim. Biophys. Acta*, **806**, 175–183. 10.1016/0005-2728(85)90094-5 2981543

[bb26] Murray, T. D., Lyubimov, A. Y., Ogata, C. M., Vo, H., Uervirojnangkoorn, M., Brunger, A. T. & Berger, J. M. (2015). *Acta Cryst.* D**71**, 1987–1997. 10.1107/S1399004715015011 PMC4601365 26457423

[bb27] Ng, J., Gavira, J. & García-Ruíz, J. M. (2003). *J. Struct. Biol.* **142**, 218–231. 10.1016/s1047-8477(03)00052-2 12718933

[bb28] Otálora, F., Gavira, J., Ng, J. D. & García-Ruiz, J. M. (2009). *Prog. Biophys. Mol. Biol.* **101**, 26–37. 10.1016/j.pbiomolbio.2009.12.004 20018206

[bb29] Pandey, S., Bean, R., Sato, T., Poudyal, I., Bielecki, J., Cruz Villarreal, J., Yefanov, O., Mariani, V., White, T. A., Kupitz, C., Hunter, M., Abdellatif, M. H., Bajt, S., Bondar, V., Echelmeier, A., Doppler, D., Emons, M., Frank, M., Fromme, R., Gevorkov, Y., Giovanetti, G., Jiang, M., Kim, D., Kim, Y., Kirkwood, H., Klimovskaia, A., Knoska, J., Koua, F. H. M., Letrun, R., Lisova, S., Maia, L., Mazalova, V., Meza, D., Michelat, T., Ourmazd, A., Palmer, G., Ramilli, M., Schubert, R., Schwander, P., Silenzi, A., Sztuk-Dambietz, J., Tolstikova, A., Chapman, H. N., Ros, A., Barty, A., Fromme, P., Mancuso, A. P. & Schmidt, M. (2020). *Nat. Methods*, **17**, 73–78. 10.1038/s41592-019-0628-z PMC9113060 31740816

[bb30] Patterson, B. D. (2014). *Crystallogr. Rev.* **20**, 242–294.

[bb31] Petsev, D. N., Chen, K., Gliko, O. & Vekilov, P. G. (2003). *Proc. Natl Acad. Sci. USA*, **100**, 792–796. 10.1073/pnas.0333065100 PMC298680 12552115

[bb32] Rosenzweig, J. B., Majernik, N., Robles, R. R., Andonian, G., Camacho, O., Fukasawa, A., Kogar, A., Lawler, G., Miao, J., Musumeci, P., Naranjo, B., Sakai, Y., Candler, R., Pound, B., Pellegrini, C., Emma, C., Halavanau, A., Hastings, J., Li, Z., Nasr, M., Tantawi, S., Anisimov, P., Carlsten, B., Krawczyk, F., Simakov, E., Faillace, L., Ferrario, M., Spataro, B., Karkare, S., Maxson, J., Ma, Y., Wurtele, J., Murokh, A., Zholents, A., Cianchi, A., Cocco, D. & van der Geer, S. B. (2020). *New J. Phys.* **22**, 093067.

[bb33] Saha, S., Özden, C., Samkutty, A., Russi, S., Cohen, A., Stratton, M. M. & Perry, S. L. (2023). *Lab Chip*, **23**, 2075–2090. 10.1039/d2lc01194h PMC10631519 36942575

[bb100] Schindelin, J., Arganda-Carreras, I., Frise, E., Kaynig, V., Longair, M., Pietzsch, T., Preibisch, S., Rueden, C., Saalfeld, S., Schmid, B., Tinevez, J. Y., White, D. J., Hartenstein, V., Eliceiri, K., Tomancak, P. & Cardona, A. (2012). *Nat. Methods*, **9**, 676–682. 10.1038/nmeth.2019 PMC3855844 22743772

[bb34] Tanaka, H., Utata, R., Tsuganezawa, K., Takahashi, S. & Tanaka, A. (2022). *Crystals*, **12**, 881.

[bb35] Tenboer, J., Basu, S., Zatsepin, N., Pande, K., Milathianaki, D., Frank, M., Hunter, M., Boutet, S., Williams, G. J., Koglin, J. E., Oberthuer, D., Heymann, M., Kupitz, C., Conrad, C., Coe, J., Roy-Chowdhury, S., Weierstall, U., James, D., Wang, D., Grant, T., Barty, A., Yefanov, O., Scales, J., Gati, C., Seuring, C., Srajer, V., Henning, R., Schwander, P., Fromme, R., Ourmazd, A., Moffat, K., Van Thor, J. J., Spence, J. C. H., Fromme, P., Chapman, H. N. & Schmidt, M. (2014). *Science*, **346**, 1242–1246. 10.1126/science.1259357 PMC4361027 25477465

[bb36] Van Aalten, D. M., Joshua-Tor, L., Crielaard, W. & Hellingwerf, K. J. (2000). *Protein Sci.* **9**, 64–72. 10.1110/ps.9.1.64 PMC2144441 10739248

[bb37] Weierstall, U., James, D., Wang, C., White, T. A., Wang, D., Liu, W., Spence, J. C. H., Bruce Doak, R., Nelson, G., Fromme, P., Fromme, R., Grotjohann, I., Kupitz, C., Zatsepin, N. A., Liu, H., Basu, S., Wacker, D., Won Han, G., Katritch, V., Boutet, S., Messerschmidt, M., Williams, G. J., Koglin, J. E., Marvin Seibert, M., Klinker, M., Gati, C., Shoeman, R. L., Barty, A., Chapman, H. N., Kirian, R. A., Beyerlein, K. R., Stevens, R. C., Li, D., Shah, S. T. A., Howe, N., Caffrey, M. & Cherezov, V. (2014). *Nat. Commun.* **5**, 3309. 10.1038/ncomms4309 PMC4061911 24525480

[bb38] Wijn, R. de, Rollet, K., Olieric, V., Hennig, O., Thome, N., Noûs, C. & Sauter, C. (2021). *J. Vis. Exp.* **169**, e61972. 10.3791/61972 33818565

[bb40] Winter, G. (2010). *J. Appl. Cryst.* **43**, 186–190.

[bb41] Winter, G., Waterman, D. G., Parkhurst, J. M., Brewster, A. S., Gildea, R. J., Gerstel, M., Fuentes-Montero, L., Vollmar, M., Michels-Clark, T., Young, I. D., Sauter, N. K. & Evans, G. (2018). *Acta Cryst.* D**74**, 85–97. 10.1107/S2059798317017235 PMC5947772 29533234

[bb42] Yu, X., Ulrich, J. & Wang, J. (2015). *Cryst. Res. Technol.* **50**, 179–187.

[bb43] Zegers, I., Carotenuto, L., Evrard, C., Garcia-Ruiz, J., De Gieter, P., Gonzales-Ramires, L., Istasse, E., Legros, J., Martial, J., Minetti, C., Otalora, F., Queeckers, P., Schockaert, C., VandeWeerdt, C., Willaert, R., Wyns, L., Yourassowsky, C. & Dubois, F. (2006). *Microgravity Sci. Technol.* **18**, 165–169.

[bb44] Zielinski, K. A., Prester, A., Andaleeb, H., Bui, S., Yefanov, O., Catapano, L., Henkel, A., Wiedorn, M. O., Lorbeer, O., Crosas, E., Meyer, J., Mariani, V., Domaracky, M., White, T. A., Fleckenstein, H., Sarrou, I., Werner, N., Betzel, C., Rohde, H., Aepfelbacher, M., Chapman, H. N., Perbandt, M., Steiner, R. A. & Oberthuer, D. (2022). *IUCrJ*, **9**, 778–791. 10.1107/S2052252522010193 PMC9634612 36381150

