# Supplementary material for: In situ counter-diffusion crystallization and long-term crystal preservation in microfluidic fixed targets for serial crystallography
Source: J Appl Crystallogr. 2024 Sep 25;57(Pt 5):1539–50. doi: 10.1107/S1600576724007544 (PMC11460377; doi:10.1107/S1600576724007544)
Supplement: Supplementary file 1 [file j-57-01539-sup1.pdf]

JOURNAL OF APPLIED CRYSTALLOGRAPHY

Volume 57 (2024)

Supporting information for article:

In situ counter-diffusion crystallization and long-term crystal preservation in microfluidic fixed targets for serial crystallography

Zhongrui Liu, Kevin Gu, Megan Shelby, Debdyuti Roy, Srinivasan Muniyappan, Marius Schmidt, Sankar Raju Narayanasamy, Matthew Coleman, Matthias Frank and Tonya Kuhl

S1. Counter Diffusion Setups

Photos and schematic figures of different counter diffusion setup designs including hydrogel reservoir, reservoir in chamber – direct, and reservoir in chamber – filter paper. In figure S1, the chip has built in counter diffusion reservoirs. This is created by shifting the channel positions on PMMA frame (layer 2, Figure 1) and cutting out square reservoirs at the edge of the sample region. The adhesive sample layer (layer 4, Figure 1) is cut to match the dimensions of the PMMA frame. Just the edge (orange arrow in Figure S1B) of the spacer layer is connected to the hydrogel reservoirs (blue dashed boxes in Figure S1B). The use of hydrogels dramatically decreases the Grashof number, so that diffusion dominates convective transport of in the chip during crystallization.

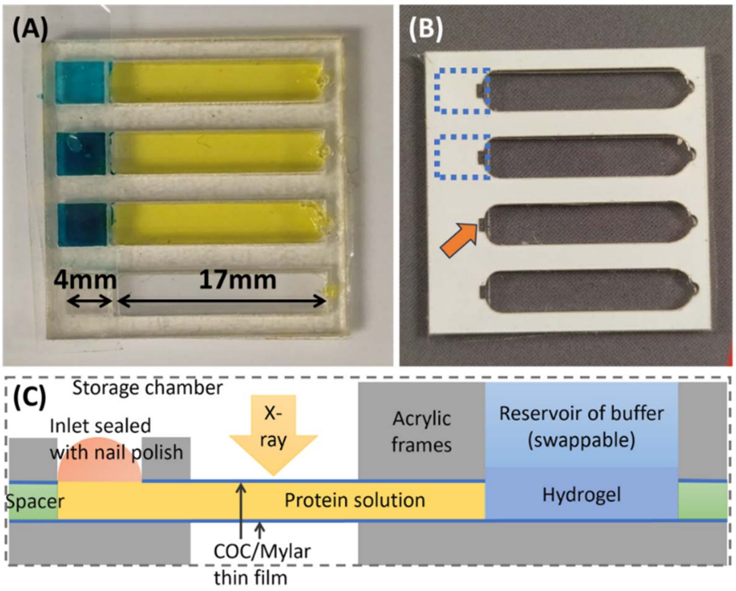

Figure S1 Photos (A, B) and schematic (C) of the hydrogel reservoir chip setup (1”×1” chip). The protein solution is loaded into the channel with a pipette, then a drop of low melting point agarose or Pluronic F127 is added to the hydrogel reservoir (A, blue) and is allowed to gel. Note, before adding buffer, the other inlet is sealed with the nail polish.

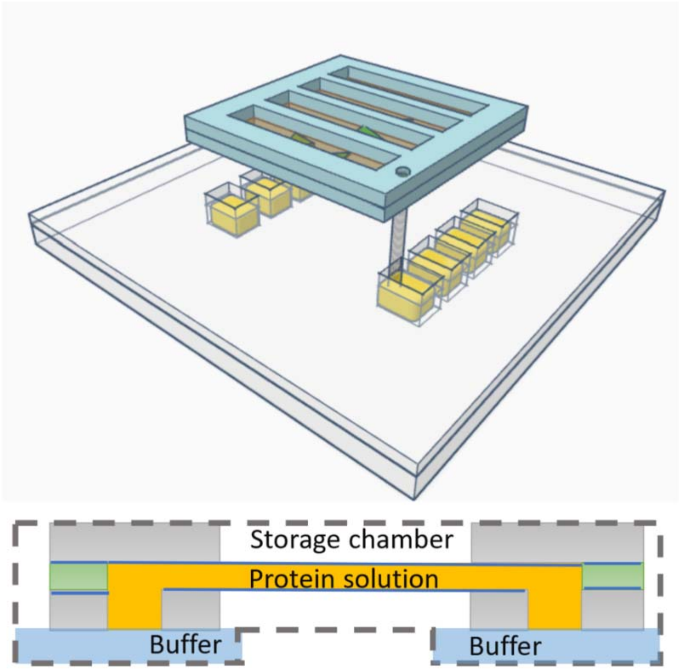

Figure S2 Reservoir in chamber – direct. In this setup the inlets were directly above the buffer reservoirs (grey rod indicating the contact location). The precipitant buffer reservoirs are fabricated within the storage chamber by cutting rectangular holes through a 2.4 mm thick piece of acrylic following the microfluidic chip dimensions. This middle sheet is glued to a 1mm thick acrylic sheet using Weld-On® 4. Separately, another layer 2.4 mm thick acrylic is cut to the outline of the microfluidic chip and glued to a 1mm acrylic piece as well. This creates an indent for the chip to reside on top of the reservoirs. Finally, a 1 mm concentric rectangular shaped silicone was inserted between the two sets of glued acrylic pieces for better sealing. The total chamber thickness is 7.8mm and crystals grown within the chip can be imagined in transmitted or reflective light with an upright or inverted microscope.

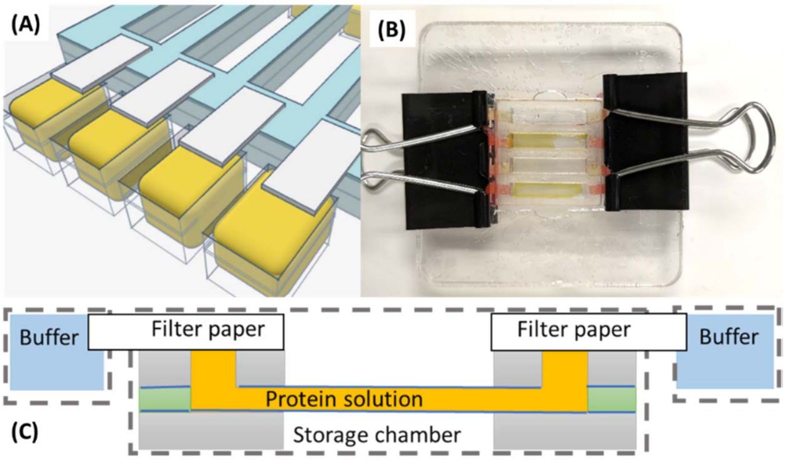

Figure S3 Reservoir in chamber – filter paper. This design is very similar to "reservoir in chamber – direct" but has filter paper strips connecting inlets to the precipitation buffer reservoir to further reduced convective flow and provided a diffusion barrier. (A) 3D CAD drawing of the reservoir and filter paper linking to the protein solution in the chip. (B) Photo of the chip inside the storage chamber. (C) Schematic of the design. This design positions the chip upright with the inlets at the top. This geometry helps prevent any leakage between the channel reservoirs or out of the inlets. After loading the precipitation buffer and placing the filter paper pieces, the setup can be sealed with a piece of Crystal Clear tape covering all the buffer reservoirs. This helps hold the filter paper pieces and prevents sample dehydration. Another acrylic piece and silicone spacer are added to the top similar to other storage chambers to seal the chamber. Binder clips or nuts and bolts can be used to seal the chamber.

S2. Fabrication of mylar and Kapton chips

Mylar and Kapton chips can be easily fabricated compared to COC chips. Since these thin films are commercially available, no spin coating or operation on wafer is required. However, these thin films are very flexible and need to be stretched taut before attaching to the supporting acrylic frames. Wrinkly film can lead to saggy windows and eventually collapsed channels that highly impacts sample loading.

To make a wrinkle-free half chip with Mylar or Kapton (Figure S4), start with carefully checking for any permanent wrinkle or damage on the film and plan for a spot-free area using an acrylic supporting frame. Next, tape the film down on all sides to a flat, clean surface. The film needs to be stretched evenly on all directions, so release the tape and repeat if wrinkles are spanning in one direction. Adhere the acrylic frame after the film is completely taut and wrinkle-free. One can adhere multiple frames on a larger film as well to speed up fabrication. Lastly, cut extra film with scalpel or sharp scissors to avoid film catching on the adhesive spacer when assembling. Puncture the thin film inside inlets as needed. A chip half is then

fabricated. Repeat this process to make the other half and assemble the halves with a piece of adhesive spacer to make a whole chip.

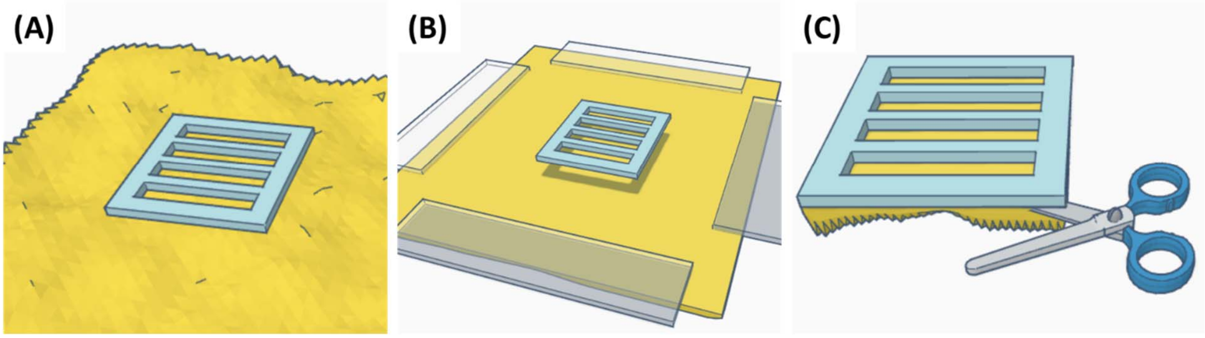

Figure S4 Fabrication steps of mylar and Kapton chip halves. (A) Cut enough film from a roll and make sure there is no permanent wrinkle or damage on the film. (B) Tape the film down to a flat, clean surface. The film needs to be stretched evenly on all directions to avoid winkles. (C) Cut extra film with scalpel or sharp scissors after adhering PMMA frame.

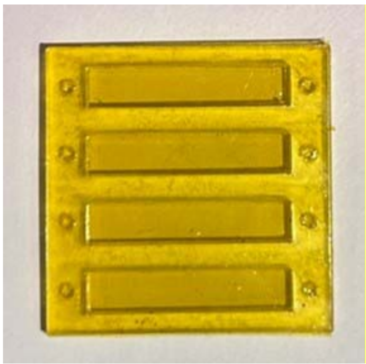

Figure S5 Photo of an assembled 1” x 1” chip with 12.7µm Kapton film windows (layer 3 in Figure 1A). The fabrication is much easier compared to 3µm Mylar films because the Kapton film is much stiffer.

S3. Crystals grown with counter diffusion setup Mylar chips

No difference in chip performance was observed when fabricating chips with different films. Figure S6 shows lysozyme crystals grown by counter diffusion in a mylar chip. Mylar window film (3µm, EW-04575-98, Spex® BoPET XRF Window Film) was purchased from Cole-Parmer.

Three independent counter diffusion crystallization trials were performed to test the different precipitation buffer conditions as well as the different counter diffusion set ups. In each of the trials, crystal size and density was measured over 4 to 5 different images frames that were approximately 3 mm by 3 mm. Each of the image frames was used to generate a data point. The figure in the main text combines all of this

data into a single data point and displays the standard deviation in both size and number of crystals (counts per frame). Figure S7 shows each of the image frames and presents the standard deviation in crystal size obtained. The number of crystals in the image frame is a discrete number.

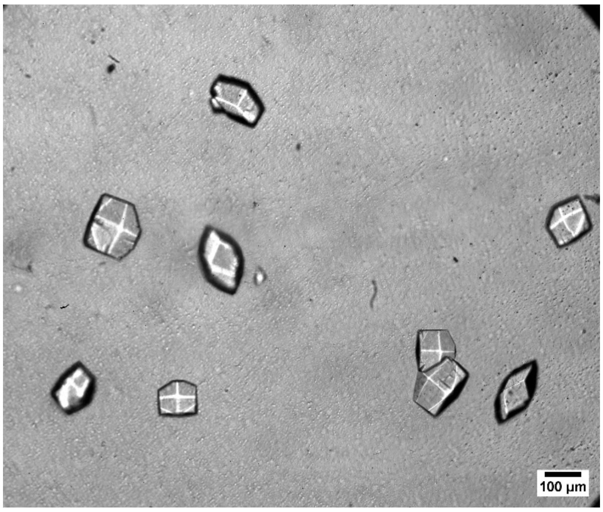

Figure S6 Lysozyme crystals grown with counter diffusion setup in a Mylar chip with 140µm channel height. The counter diffusion results for any design are very similar between COC chips and Mylar chips. The texture in the background came from the Mylar films.

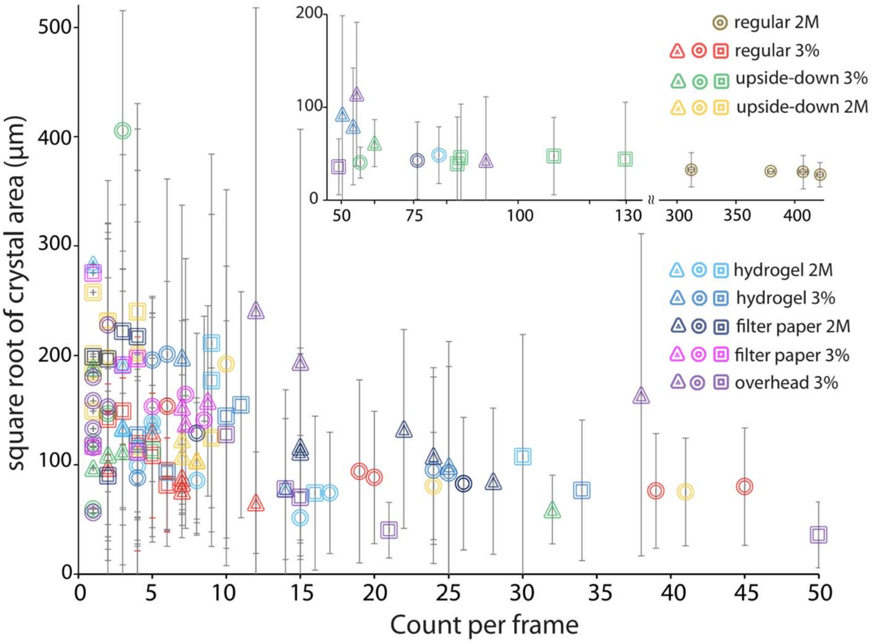

Figure S7 Crystal size and density obtained using different crystallization conditions. Data points of the same color but different symbols (△ ◎ ▣) represent distinct experimental trials conducted under the

same conditions of protein and precipitant buffer. At least 4 different image frames were taken per trial. Thus, there are multiple symbols for each trial that represent different image frames (approximately 3mm x 3mm images) taken on the same chip. The error bars represent the variation in crystal size in each image frame and the number of crystals per frame (counts per frame). Different crystallization conditions were used; “2M” stands for 2 M NaCl with 0.1 M sodium acetate buffer pH 4.6, which yield regular sized (~30µm) crystals in micro batch; “3%” stands for 3% w/v NaCl in 0.5M tris HCl pH8.5 – conditions that typically yield larger (~80µm) crystals in micro batch; “regular” stands for typical micro batch conditions in a microfluidic chip. These micro batch conditions were done with a 1:1 mixture of protein solution and precipitating buffer in the channels. Counter diffusion setups included “upside-down” for “upside-down chip in chamber” where the chip was placed upside down in a petri dish and contacted with a reservoir of precipitant buffer; “hydrogel” stands for 30% Pluronic F127 hydrogel reservoir chip; “overhead” and “filter paper” stands for reservoir in a storage chamber with direct contact with the precipitant buffer or indirect contact through filter paper, respectively.

S4. Microscopic images of photosystem I crystal slurry in storage chamber.

To demonstrate the stability of chips in the storage chambers, the Fromme group at ASU fabricated a mylar chip and loaded a slurry of photosystem 1 (PS1) crystals using a standard pipette with 4.5 µl per channel. PS1 is crystallized in low salt conditions and very sensitive to dehydration as the PS1 crystals will dissolve when the salt concentration increases. Figure S8 shows PS1 crystals.

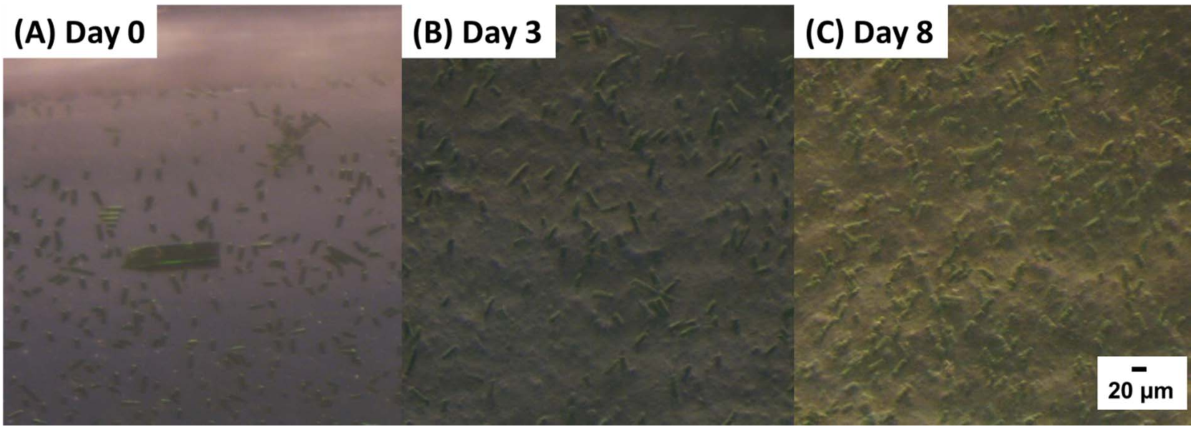

Figure S8 A slurry of photosystem I crystals was loaded into a just assembled mylar chip. Afterwards, the sample loaded chip was placed in an acrylic storage chamber and left at ambient conditions. (A) Images of the crystals were taken on day 0 after loading; (B) at day 3; (C) day 8. The images were provided by Petra Fromme lab who independently tested the performance of the chip and the storage

chamber. The photosystem I crystals are very susceptible to dehydration and will disintegrate within 10 minutes under ambient conditions.

S5. Microscopic image of bovine liver catalase crystals obtained through counter diffusion in the customized storage chamber.

Figure S9 is an image of bovine liver catalase grown in the chip using counter diffusion. The protein solution was 40 mg/mL in 50 mM sodium phosphate buffer, pH 6.8. The precipitation buffer was 22.5% PEG 4000 in 0.1 M Tris buffer, pH 8.5. In micro batch conditions of a 50:50 protein to precipitation buffer, the protein crystals average about 70 µm compared to 100s of µm using counter diffusion (Gilbile et al., 2021).

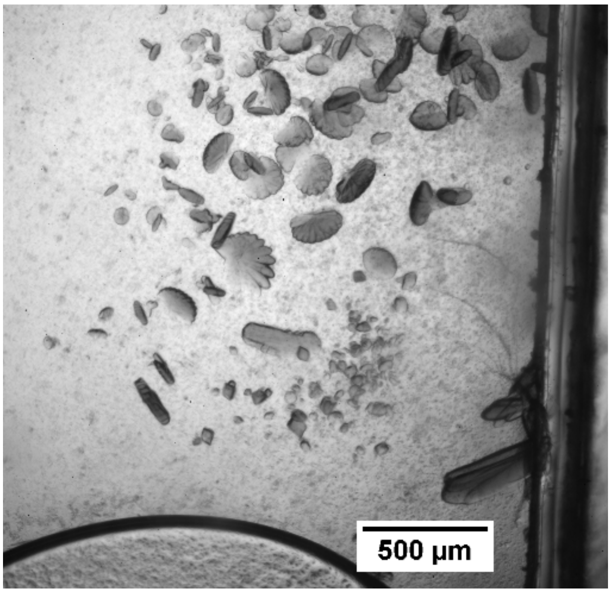

Figure S9 Bovine liver catalase crystals obtained through counter diffusion in the storage chamber (upside-down chip in chamber). Crystals are typically ~70µm in a micro batch experiment with the same conditions.
